# Supercritical CO_2_ Extraction vs. Hexane Extraction and Cold Pressing: Comparative Analysis of Seed Oils from Six Plant Species

**DOI:** 10.3390/plants13233409

**Published:** 2024-12-04

**Authors:** Katja Schoss, Nina Kočevar Glavač

**Affiliations:** Department of Pharmaceutical Biology, Faculty of Pharmacy, University of Ljubljana, SI-1000 Ljubljana, Slovenia; nina.kocevar.glavac@ffa.uni-lj.si

**Keywords:** cold pressing, CO_2_ extraction, solvent extraction, plant seed oils, unsaponifiable matter

## Abstract

Supercritical fluid extraction using carbon dioxide (SFE-CO_2_) brings a convincing advance in the production of plant oils used in cosmetics, in fortified foods and dietary supplements, and in pharmaceuticals and medicine. The SFE-CO_2_-extracted, hexane-extracted, and cold-pressed plant oils of pumpkin (*Cucurbita pepo* L.), flax (*Linum usitatissimum* L.), linden (*Tilia* sp.), poppy (*Papaver somniferum* L.), apricot (*Prunus armeniaca* L.), and marigold (*Calendula officinalis* L.) seeds were investigated in terms of oil yield, fatty acid composition, unsaponifiable matter yield and composition, and the antioxidant activity of unsaponifiable matter. SFE-CO_2_ proved to be the preferred extraction method for four out of six plant materials, especially for seeds with lower oil content. However, for seeds with higher oil content, such as apricots, cold pressing is a viable alternative. A comparison of fatty acid composition did not reveal significant differences between extraction techniques. SFE-CO_2_ extraction improved the total phytosterol content of oils, especially pumpkin seed oil. A high variability in the antioxidant potential of the unsaponifiable matter studied was determined, with pumpkin seed oil showing the highest antioxidant activity. A correlation analysis was performed between unsaponifiable composition and antioxidant activity, and showed statistically significant correlations with squalene, cycloartenol, and an unidentified compound. This is the first comparison of the phytosterol compositions of linseed, apricot, linden, and marigold. Through continued optimization, SFE-CO_2_ has the potential to revolutionize the production of plant oils and provide a sustainable and efficient alternative.

## 1. Introduction

Plant oils contain essential nutrients, which is one of the reasons that they play an important role in the food industry and are a vital part of human nutrition [1]. They are also widely used in the pharmaceutical and cosmetic industries as active ingredients, lipid vehicles, emollients, and other purposes [2]. However, the suitability of an oil for a specific purpose is determined by its characteristics, fatty acid composition, and the amount and composition of unsaponifiable matter.

The most commonly used plant oil extraction methods are cold pressing and extraction using organic solvents, typically hexane. Solvent extraction is an efficient method, with an average recovery of up to 99% of available oil [3]. The disadvantages of this process are high costs, solvent residues in the obtained oil and the associated toxicity risks, and ecological problems due to the losses of organic solvents into the environment. Although hexane as a solvent is allowed in the food industry for specific purposes by the Food and Drug Administration (FDA) [4] and the European Commission [5], it is classified as hazardous and as a non-preferred solvent. For this reason, other toxicologically less-invasive organic solvents, such as ethanol and isohexane, have been researched for lipid extraction [6,7]. Cold pressing is another preferred method to hexane extraction due to the absence of solvent use and thus lower costs, though it has a significant limitation, i.e., a low oil yield [3].

To obtain the highest possible amount of lipids from plant seeds, due to the higher penetration power of supercritical fluids into porous solid materials, supercritical fluid extraction (SFE) has been gaining attention on an industrial level since the beginning of the 20th century as a novel technique that avoids all of the above-described difficulties. SFE is environmentally friendly as no organic solvents are needed. Carbon dioxide (CO_2_) is the most commonly used supercritical solvent because it has many advantages: it is inflammable, non-toxic, and easily available at a low price, and it can be recycled after extraction, reducing overall energy consumption and increasing the sustainability of the process. An additional advantage of SFE is the FDA’s green light for the unrestricted use of supercritical extracts in the food industry [8,9,10].

Chemically speaking, plant oils are a mixture of triglycerides (98–99%) and unsaponifiable compounds. Triglycerides are esters of glycerol and fatty acids (C10–20), which can be saturated, monounsaturated, or polyunsaturated, each influencing the oil’s stability and health benefits [11]. Triglycerides are responsible for the physical and chemical properties, as well as the biological effects, of butters and oils, such as antimicrobial, anti-inflammatory, and regenerative activities [11]. Significantly less research has been performed on unsaponifiable compounds, which usually represent between 0.3 and 2% of a plant oil [11,12]. The unsaponifiable fraction is a complex mixture of phytosterols, waxes, tocopherols and tocotrienols, phospholipids, and terpenic and phenolic compounds and is very important for the investigation of the quality of plant oils [11,12,13]. Unsaponifiable compounds have been shown in vitro to act as antioxidant, anti-inflammatory, antitumor, immunomodulatory, and antimicrobial agents, to have antinociceptive effects, and to promote wound healing [13,14]. Phytosterols, in particular, are structurally and functionally similar to cholesterol and have been shown to reduce the levels of reactive forms of oxygen [15], help lower blood cholesterol levels [16,17,18], and reduce the risk of cardiovascular disease in humans when consumed [18,19]. Currently, phytosterols extracted from various plant sources are commonly used in fortified foods and dietary supplements [18].

With the progress of research and knowledge, plant oils and their structural compounds have been intensively researched in recent decades for their useful applications in many areas. At the same time, a number of methods for their extraction have been established, while it is a well-known fact that different methods of oil extraction lead to different compositions of the oils. The final use of an oil depends on the composition or properties of the oil. Thus, knowing the exact composition based on the method selected can lead to the choice of the best method of oil extraction at an industrial level, taking into account the intended use to achieve the desired biological effect (therapeutic, cosmetic, or nutritional). This study is, to the best of our knowledge, the first study that attempts a detailed analysis of the fatty acid and unsaponifiable matter composition of selected plant seed oils obtained using different extraction methods: hexane extraction, cold pressing and SFE with CO_2_ (SFE-CO_2_). The oil yield, the proportion of unsaponifiable matter, and the in vitro antioxidant activity of the obtained unsaponifiable matter were also determined and correlated with published reports to contribute to the establishment of a global database of plant oils from the seeds of pumpkin (*Cucurbita pepo* L.), flax (*Linum usitatissimum* L.), linden (*Tilia* sp.), poppy (*Papaver somniferum* L.), apricot (*Prunus armeniaca* L.), and marigold (*Calendula officinalis* L.). These plants were chosen for their diverse oil compositions, industrial relevance, and potential health benefits. Pumpkin and apricot seeds, for instance, are valued for their high oil content, while linden and marigold seeds are rich in unsaponifiable matter despite their lower yields. By comparing cold pressing, solvent extraction, and SFE, we aim to evaluate the efficacy and sustainability of these methods in extracting oils with high nutritional and industrial value.

## 2. Results and Discussion

Hexane extraction is the most common method for the production of plant oils. It was therefore selected as a reference method for obtaining oils in this research. The disadvantage of such extraction, as mentioned in the introductory section, is the residue of hexane, which is a chemical of health concern. It is, for example, hepatotoxic in rodents [20,21], and higher long-term exposure leads to polyneuropathy in humans [22,23]. Furthermore, it is an environmental pollutant. Under the 1990 Clean Air Act, it is considered to be an ozone precursor, is classified as a hazardous air pollutant [24], and is identified as toxic to reproduction by the European Chemical Agency (ECHA) [25]. In addition, prolonged heating during the evaporation of hexane causes the thermal degradation of oils [26].

Thus, alternative techniques have been intensively explored. In this study, ethanol was tested as an alternative green organic solvent, as suggested by Castejòn et al. [7]. Other methods used were methods where plant oils are obtained without the use of organic solvents: SFE-CO_2_ and the most ancient method—cold pressing.

### 2.1. Oil Yield

The oil yield varied considerably depending on the extraction method used (Table 1). Cold pressing proved to be suboptimal for the selected plant seeds in our study, with minimum extraction yields ranging from 10 to 25%. SFE-CO_2_ proved to be the preferred extraction method for four out of six plant materials, especially for plant drugs with small seeds (such as linseed, linden, poppy, and marigold) and seeds with lower oil content (linden and marigold). When planning SFE-CO_2_, however, one must take account of the fact that the oil yield depends on numerous factors, including pressure, temperature, time, CO_2_ flow rate, and the particle size and moisture content of the oil-bearing material. In extensive seed oil processing studies, SFE-CO_2_ has also been shown to be superior to solvent extraction and cold pressing for a variety of seed types [26,27]. Additionally, in the case of pumpkin seed oil, a difference in the color of the oils was evident: the oil extracted with hexane was brownish-green and the oil extracted using SFE-CO_2_ was orange-yellow. The latter was clearer, which can also be explained by the lower selectivity of extraction with organic solvents and the associated extraction of other compounds from the seeds, as was also noted by Hrabovski et al. [28]. It is also noteworthy that the yield of linseed oil was lower than reported in the literature, potentially indicating issues with seed quality or moisture content.

Conversely, hexane extraction proved to be a superior extraction method for pumpkin and apricot seeds. This was also shown in the re-extraction (hexane, 22 °C) of the seed meal for apricot seeds, where 11.9% of the oil was re-extracted with hexane after SFE-CO_2_. For the other seed meals, low re-extraction yields were obtained: 4.4% from flax seeds, 4.0% from poppy seeds, 3.2% from pumpkin seeds, 1.2% from linden seeds, and 1.0% from marigold seeds. In the oil industry, solvent extraction is usually also carried out after cold pressing in order to recover residual oil, particularly for low-oil seed crops [26]. Residual oils were also analyzed for their fatty acid composition, but no differences were found compared to hexane-extracted oils. Table 1 also shows that temperature (22 °C and 55 °C) did not affect oil yield significantly.

In our study, acetone and ethanol were tested in addition to hexane, with varying drug-to-solvent ratios (1:3 and 1:10) and temperatures (22 °C and 55 °C). For an efficient solvent extraction, it is important that the solvent can dissolve and extract the oils from the seeds [29]. Although alcohols (e.g., methanol, ethanol, and isopropanol) were originally favored as an alternative to hexane, particularly for their ability to extract oils and fats [7,26], their use is associated with significant limiting problems. The most significant are the poor solubility of oils in alcohols and an extremely non-selective extraction, as polar compounds are co-extracted to a large extent. In addition, the obtained crude oil still must undergo additional purification steps, leading to high production costs. In our oil samples, notable differences in consistency were observed between oils extracted using hexane and ethanol, with the latter appearing more viscous and lumpier with a dark green color, showing no similarity to oil. This observation is consistent with the tendency of ethanol to dissolve chlorophyll, which is particularly abundant in pumpkin seeds [30].

### 2.2. Fatty Acid Composition

This is the first research comparing CO_2_, hexane, and mechanical extraction in terms of fatty acid composition for pumpkin, linden, marigold, and apricot seeds. Overall, the analysis of the fatty acid composition of hexane extracts in comparison to SFE-CO_2_ C-V1, the main oil-containing CO_2_ extract, and cold-pressed oil did not reveal significant differences, as shown in Table 2. The main components of the individual plant seed oils are consistent with their typical compositions and natural variations, as expected, which proves the suitability of all oil extraction methods for the production of oils in terms of fatty acid composition [26,31,32]. It is worth emphasizing, however, that SFE-CO_2_ can be adapted for the targeted extraction of triglycerides. Previous studies have shown that it is capable of selectively extracting triglycerides with polyunsaturated fatty acids [26]. An overview of the composition of oil samples is given below for each oil separately.

Pumpkin seed oil. In the oil’s fatty acid composition, oleic acid (43.6–45.5%) was predominant, followed by linoleic acid (34.6–35.4%), palmitic acid (9.8–11.1%), and stearic acid (6.9–8.8%), which corresponds to typical composition [33]. Differences were observed when comparing fractions obtained using SFE-CO_2_. In particular, the C-V1 fraction, the main oil-rich fraction, had a slightly lower level of stearic acid (7.6%) and a slightly higher level of oleic acid (45.5%) than the C-V3 fraction (9.1% and 37.9%, respectively), which was not surprising in terms of stearic and oleic acid contents in hexane extracts since the solubility of stearic acid has been found to be lower in supercritical CO_2_ [26].

Interestingly, the C-V3 fraction yielded two immiscible phases: one polar and one non-polar. The polar phase was excluded from the SFE-CO_2_ yield. The non-polar C-V3 phase contained more saturated fatty acids (24.9% compared with 18.4% in C-V1), more palmitic acid (15.3% compared with 10.4% in C-V1), and less linoleic acid (32.6% compared with 34.6% in C-V1) and oleic acid (37.9% compared with 45.5% in C-V1). The GC–MS analysis of the C-V3 polar phase revealed the presence of two compounds: 2,3-butadienol (relative area of 98.6%) and an unidentified compound (*m*/*z*: 277 (100) 278 (39) 77 (34)) with a relative area of 1.4%. The detection of 2,3-butadienol is consistent with previous studies investigating volatile compounds in cold-pressed pumpkin seed oils that contribute to its specific odor [34].

Linseed oil. The fatty acid composition of linseed oils was similar for the different extraction methods. α-linolenic acid dominated the composition and accounted for 73.0–75.1% of the total fatty acids, followed by linoleic acid at 14.0–14.8% and palmitic acid at 5.4–5.7%, while other fatty acids were present in proportions of less than 5%. The results were consistent with the typical composition of linseed oil, with one notable exception: oleic acid content, which is normally in the range of 10–22% [11,27,35], but was absent in our samples. This is most likely due to the origin of the seeds.

Comparative analyses between SFE-CO_2_ and organic solvent extraction methods have been performed in previous studies that generally correspond to the results of our study. Bozan et al. [36] reported a 4% higher α-linolenic acid content in oils extracted with SFE-CO_2_ compared to oils obtained by solvent extraction using petroleum ether. Similarly, Pradhan et al. [27] investigated the extraction of linseed oil using SFE-CO_2_ and compared it with hexane extraction and cold-pressing methods. Their results showed that SFE-CO_2_ selectively extracted fatty oils with higher percentages of ɷ−3 (55%) and ɷ−6 (16%) fatty acids.

Linden seed oil. Linden seeds are an uncommon yet noteworthy source of plant oil that is characterized by a special triglyceride composition with uncommon cyclopropenoic fatty acids that typically range between 6 and 17% [37,38]. Looking more closely at the typical fatty acid composition reported in rare previous studies, linoleic acid content is typically between 41 and 62%, oleic acid content between 14 and 24%, palmitic acid content between 7.5 and 10%, *cis*-malvalic acid content between 1 and 10%, and *cis*-sterculic acid content between 0.3 and 11% [37,38]. This is in line with the fatty acid profile observed in this study: linoleic acid accounted for the highest proportion at 54.2–54.9%, followed by oleic acid (21.4–23.1%), palmitic acid (7.3–8.0%), and malvalic acid (5.3–6.0%). The differences in fatty acid composition between all extracted linden seed oils were therefore not significant.

Poppy seed oil. The fatty acid composition of all triglycerides, except SFE-CO_2_ C-V3, showed no noteworthy differences. In all cases, the predominant fatty acid was linoleic acid, which accounted for 68.1–68.9% of total fatty acids, followed by oleic acid (18.3–19.1%) and palmitic acid (7.5–8.8%). These results are in line with the typical composition of poppy seed oil, as described in the previous literature [11,39,40,41]. In C-V3, a notably higher percentage of palmitic acid (12.4% vs compared with 7.5% in C-V1) and lower percentage of linoleic acid (54.1% compared with 68.5% in C-V1) were observed. This observation was similar to the C-V1 and C-V3 pumpkin seed extracts. Therefore, a significantly higher difference in total saturated fatty acids and total unsaturated fatty acids was observed between C-V1 and C-V3 in poppy seed oils.

Previous studies have performed comparative compositional analyses between SFE-CO_2_ and organic solvent extraction methods and confirmed that SFE-CO_2_ yields oils of comparable composition to those obtained by cold pressing or solvent extraction under parameters similar to ours. However, Bozan and Temelli [42] observed significant differences in all fatty acids, except stearic acid, between oils extracted at 50 °C and those extracted at 70 °C using SFE-CO_2_ and solvent extraction. In particular, they observed a decrease in oleic acid content with increasing temperature, accompanied by an increase in linoleic acid content. In addition, Dąbrowski et al. [43] investigated the effects of the different polar modifiers of SFE-CO_2_ (acetone, ethanol, ethyl acetate) compared to SFE using pure CO_2_, Soxhlet extraction using *n*-hexane, and cold pressing. The fatty acid composition remained the same regardless of the extraction method, which is fully consistent with our results.

Apricot kernel oil. All extracted apricot oils showed a similar composition, with oleic acid being the predominant component of triglycerides (68.3–72.3%), followed by linoleic acid (20.0–22.8%). Other fatty acids were only present to a small extent and together accounted for less than 5%. These results are consistent with the typical composition of cold-pressed apricot oil reported in previous studies [44], while no studies on SFE-CO_2_ were found in the literature reviewed.

Marigold seed oil. Although marigold seeds are not usually selected for oil extraction, they have a notable oil content of about 20% maximum [45]. Marigold seed oil has an atypical fatty acid composition. A significant proportion (around 60%) consists of calendic acid, which is characterized by a rare conjugated structure (8*t*,10*t*,12*c*−18:3) and is biosynthesized in marigold through the desaturation of linoleic acid. The percentage of calendic acid was found to correlate closely with the ripening stage of the seeds, increasing from 8.62% to 53% during the ripening period (0–2 weeks after flower drop), accompanied by a decrease in the content of linoleic and oleic acids [45]. In our study, calendic acid accounted for the highest proportion in all marigold seed oil samples, ranging from 56.3–62.2%. Two SFE-CO_2_ fractions were obtained: C-V1 had a higher percentage of calendic acid at 62.2% than C-V2 at 58.9%. In addition, the samples contained linoleic acid (25.8–28.7%) and oleic acid (4.3–6.1%), while other fatty acids were present at less than 5%. Overall, the composition was consistent with the typical fatty acid profile of marigold seed oil from ripe seeds [45].

### 2.3. Unsaponifiable Matter—Yield and Composition

Although SFE-CO_2_ is recognized as a fast-growing method for oil extraction, only a few studies have been published comparing the yield and composition of unsaponifiable matter obtained using SFE-CO_2_ vs. cold pressing or hexane extraction [17]. In this study, the yield of unsaponifiable matter and the content of phytosterols and squalene were analyzed in cold-pressed and SFE-CO_2_-extracted oils (Table 3). It should be emphasized that this is the first comparison between the phytosterol compositions of linseed, apricot, linden, and marigold seed oils.

The yield of unsaponifiable matter was generally between 0.7 and 2.2% and was the lowest in poppy and apricot oils. The values are within the normal range of 0.3 to 2% of the oil, with slight variations between the different plants [13]. However, for all six plant oils, the yield of unsaponifiable matter was higher in SFE-CO_2_-extracted oils (fractions C-V1, C-V2, C-V3) than in cold-pressed oils and was generally higher in C-V2 and C-V3 than in C-V1. The largest difference was observed in pumpkin seed oils (cold-pressed: 1.1%, SFE-CO_2_ C-V3: 4.0 %). Our conclusion is that SFE-CO_2_ facilitates a higher-than-average yield of unsaponifiable matter in the oil. The method can be further improved to increase the yield of unsaponifiable matter through additional modifications of extraction parameters by adding ethanol as a polar co-solvent, as previously suggested by other authors [17,46]. This is because phytosterols are slightly polar (due to the hydroxyl group in carbon 3) [17].

The composition of unsaponifiable matter is very diverse and consists of different groups of molecules (e.g., phenols, squalene, carotenoids, tocopherols, and phytosterols) [13], with phytosterols generally being the most abundant. To date, more than 200 types of phytosterols have been discovered, of which β-sitosterol, campesterol, and stigmasterol are the most abundant [19,46]. In nature, phytosterols occur as free sterols or as conjugates of fatty acid esters, glycosides, and acetylated glycosides [18].

Due to the growing demand for isolated phytosterols, which have been used increasingly in cosmetics, fortified foods, and dietary supplements [17,19], different isolation techniques have been developed, depending on the type of plant matrix and the form of phytosterols [18]. The use of phytosterols obtained using SFE-CO_2_ is attractive due to the solvent-free products and environmentally friendly processing technology [19,26]. However, phytosterols are mainly located in the cell membrane, so the phytosterol extraction process in longer than the oil extraction process [17].

In existing studies on unsaponifiable matter, the content of individual compounds is most frequently given as a proportion (in %), i.e., as a percentage of the relative peak area of an individual compound in a GC–MS chromatogram. The relative peak areas of the investigated unsaponifiable compounds are listed in Table 3, which served as our bases for the interpretation of the results and comparison with data from the literature. However, it should be noted that these values do not accurately reflect the actual content of the compounds present in the unsaponifiable matter samples. Therefore, selected compounds, namely, squalene, β-sitosterol, campesterol, cholesterol, and stigmasterol, were quantified using reference compounds and are listed in Table 4 and expressed in mg/g oil. These values were determined by using calibration curves. It must be emphasized that some results (e.g., squalane and β-sitosterol in pumpkin seed oil) indicate that the relative peak areas (in %) cannot be considered a criterion for predicting the amount of phytosterols. For details, please see the discussion below.

Pumpkin seed oil. Six components were identified in the unsaponifiable fraction of pumpkin seed oil, with β-sitosterol being the most abundant and present in all samples in varying amounts (2.58–2.83 mg/g oil, 12.3−23.7%), with the highest concentration observed in C-V3. Squalene had the highest proportion (29.5−76.9%), but a low content (0.05–0.13 mg/g oil). An interesting component of the pumpkin seed oil unsaponifiable matter is cycloartenol, metabolically the first precursor of phytosterols, which was detected in pumpkin and linden seed oil unsaponifiable matter only. It had the highest percentage in cold-pressed oil (12.3%). Previous studies also found the presence of γ-tocopherol, squalene, β-sitosterol, and stigmasterol in the unsaponifiable fraction of pumpkin seed oil [47,48], which is consistent with our results. In another study by Habrovsky et al. [28], the phytosterols of different extraction methods were compared, with extraction using the organic solvents hexane and petroleum ether and using supercritical carbon dioxide at 400 bar and 40 °C. The oil yield was 43%, 45%, and 36% for extraction with hexane, petroleum ether, and SFE-CO_2_, respectively, while the total phytosterol content in the SFE-CO_2_ extract (294 mg/100 g oil) was about 30% higher than in the hexane extract and about 20% higher than in the petroleum ether extract.

Linseed oil. Seven components were detected in the unsaponifiable matter of linseed oil, with β-sitosterol being the most abundant (32.9% in the cold-pressed sample or 2.90 mg/g and 20.1% in the SFE-CO_2_-extracted sample or 2.68 mg/g) followed by 9,19-cyclolanost-24-en-3-ol (26.1% in the cold-pressed sample and 22.1% in the SFE-CO_2_-extracted sample), and campesterol (11.9% or 2.44 mg/mL in the cold-pressed sample and 13.9% or 2.18 mg/mL in the SFE-CO_2_-extracted sample). The high proportions of β-sitosterol and campesterol are in line with previous research findings. Additionally, cholesterol, clerostenol, brassicasterol, campestanol, cycloartenol, and various forms of avenasterol were detected in linseed oil [49,50]. The composition of these components varies, depending on the type and origin and maturation of the seeds.

Linden seed oil. The composition of unsaponifiable compounds in linden seed oil was generally similar in both extraction methods, with β-sitosterol being the most abundant, particularly in the cold-pressed sample (67% or 4.20 mg/g in the cold-pressed sample and 81% or 2.68 mg/g in the SFE-CO_2_-extracted sample), followed by campesterol, stigmasterol, and squalene. The cold-pressed sample also contained a low proportion of cycloartenol (1.4%). According to the literature, linden seed oil exhibits a high proportion of phytosterols, with β-sitosterol being the dominant compound, which is consistent with our findings. In addition to the previously mentioned compounds, linden seed oil is expected to contain Δ5-stigmasterol and Δ5-avenasterol, which were not found in our samples [51].

Poppy seed oil. The unsaponifiable matter of poppy seed oil had the highest β-sitosterol content (41–54% or 2.71–3.73 mg/g), followed by squalene, campesterol, stigmasterol, cholest-7-en-3-ol, and desmosterol. A study by Dąbrowski et al. [43] also analyzed poppy seed oils obtained through different extraction methods (cold pressing, Soxhlet extraction with *n*-hexane and SFE-CO_2_), and demonstrated that these methods influenced the composition of the unsaponifiable fraction. It was found that the main group comprised phytosterols, with content ranging from 2.52 mg/g (cold-pressed oil) to 2.93 mg/g (oil extracted by SFE-CO_2_). The main representative of phytosterols was β-sitosterol with 1.84–2.13 mg/g (approx. 63% of total phytosterol content). The greatest variation in compound content according to the method of extraction was observed for cycloartenol (coefficient of variation of 23.1%), for which the mass share in the final oil varied from 24.6 mg/kg of oil (cold-pressed oil) to 42.1 mg/kg of oil (oil extracted by SFE-CO_2_). However, the proportion of cycloartenol in total poppy seed sterols was low and did not exceed 1.4%.

Apricot kernel oil. In the unsaponifiable matter of apricot oil, β-sitosterol was also the most abundant (62−90% or 3.67–4.28 mg/g), followed by campesterol, squalene, and γ-tocopherol. Other research on the unsaponifiable matter of apricot oil has reported similar findings. Campesterol and β-sitosterol were found in the highest proportions among sterols, representing up to 80% of the sterol content, which is consistent with our results. Cholesterol, which was not present in our samples, was also detected in their analysis. Squalene was discovered in the same study, marking the first reported occurrence of squalene in apricot oil. Another study reported a high proportion of phytosterols and identified four tocopherol isomers, with γ-tocopherol being the most abundant [52,53].

Marigold seed oil. A total of 12 components were identified in the unsaponifiable matter of marigold seed oil, with β-sitosterol once again being the most abundant (33−57% or 2.69–3.07 mg/g). In the unsaponifiable part of cold-pressed oil, however, stigmasterol had a higher content (3.43 mg/g) than β-sitosterol (2.81 mg/g). Notably, fractions C-V1 and C-V2 exhibited differences in the composition of the unsaponifiable matter. C-V1 did not contain squalene and cholesterol, whereas these two components constituted less than two percent in C-V2. C-V2 demonstrated one of the highest proportions of γ-tocopherol among all the investigated oils, at 18.8%. To the best of our knowledge, this is the first analysis of the unsaponifiable matter of marigold seed oil conducted.

### 2.4. Unsaponifiable Matter—Antioxidant Activity

Compounds with antioxidant activity are an essential part of unsaponifiable matter and contribute significantly to the overall stability and biological effects of plant oils. In the cosmetics industry, oils obtained using SFE-CO_2_ are often advertised as having increased oxidative stability. However, a direct correlation between the oil extraction method, oil composition, and antioxidant activity has not yet been proven, as only a few studies have been conducted on this topic. It is therefore essential to evaluate the antioxidant activity of each component, including the unsaponifiable compounds, in each oil and for each extraction method separately in order to determine the most beneficial process.

**Figure 1:**
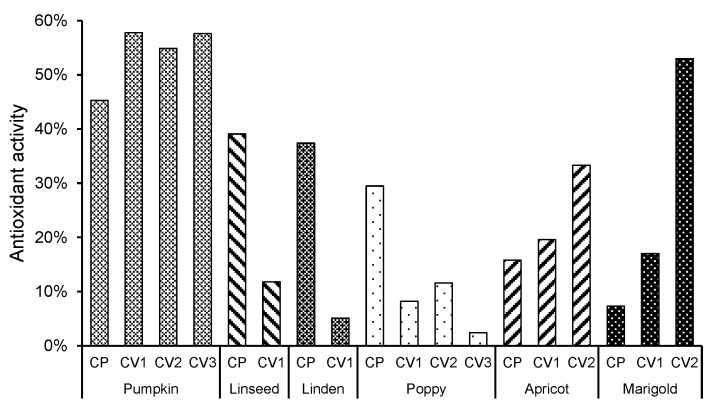
Antioxidant activity of unsaponifiable matter from plant seed oils extracted using different methods. Results are presented for unsaponifiable matter obtained from oils extracted by cold pressing (CP) and supercritical CO_2_ extraction (C-V1, C-V2, C-V3). Antioxidant activity was assessed using the DPPH assay and is expressed as a percentage (%) of free radical scavenging capacity.

Antioxidant activity was assessed using the DPPH assay, which measures the ability of the unsaponifiable matter to scavenge free radicals. Results are expressed as a percentage of free radical scavenging capacity, where a higher percentage indicates stronger antioxidant activity. The results showed a high variability in the antioxidant activity of the unsaponifiable matter studied (Figure 1). The most noteworthy was the antioxidant activity of all pumpkin seed oil samples (antioxidant activity of between 39% and 50%) and the C-V2 fraction of marigold seed oil (57%). In pumpkin, apricot, and marigold seed oils, the antioxidant activity was higher in SFE-CO_2_-extracted samples, while, interestingly, the antioxidant activity of linseed, linden, and poppy unsaponifiable matter was higher in cold-pressed oil. A correlation analysis was performed between the composition of unsaponifiable compounds and antioxidant activity, which showed statistically significant correlations (*p* = 0.05) with squalene (0.677), cycloartenol (0.622), and an unidentified compound (*m*/*z*: 343 (100), 75 (57), 55 (52), 73 (46)) at 41.39 min (0.510). Samples with higher concentrations of these three components showed higher antioxidant activity.

In reviewing the literature, we found only one study comparing the antioxidant activity of unsaponifiable matter obtained by different methods. That study was performed on poppy seed oil. Bozan and Temeli [42] compared extractions using SFE-CO_2_ and petroleum ether, but they evaluated the content of tocols, which were not detected in our analysis (with the exception of γ-tocopherol), and no other unsaponifiable compounds.

## 3. Materials and Methods

### 3.1. Chemicals

DMSO (dimethyl sulphoxide), DPPH (2,2-diphenyl-1-picrylhydrazyl), KOH, and phenolphthalein were obtained from Sigma-Aldrich^®^, Prague, Czech Republic; diethyl ether, ethanol (99% and 96% purity), *n*-hexane, and methanol were obtained from Carlo-Erba Reagents, Val-de-Reuil, France; silylating mixture I by Supelco was obtained from Merck, Darmstadt, Germany; and HCl (37%) was obtained from J.T. Baker, Darmstadt, Germany. The *n*-hexane Suprasolv^®^ used for GC–MS was of GC–MS-grade purity and was obtained from Merck, Darmstadt, Germany. Reference standard compounds that were used for the confirmation of identity and quantification on GC–MS were from the following suppliers: F.A.M.E. mix C4-C24 from Supelco, Bellefonte, PA, USA; β-sitosterol and stigmasterol from Carl Roth, Germany; cholesterol from Fluka, Heidelberg, Germany; and campesterol from PhytoLab, Vestenbergsgreuth, Germany.

### 3.2. Plant Materials and Oil Preparation

The study was conducted on six plant seeds (Table 5). Before extraction, seeds were ground into powder using a laboratory mill (IKA A10, Staufen, Germany) to achieve a particle size smaller than 0.5 mm.

### 3.3. Oil Extraction

#### 3.3.1. Ultrasound-Assisted Extraction

Oil was extracted according to the technique described by Castejón et al. [7] with slight modifications. Dried plant seeds were ground (5 g), mixed with an organic solvent (ethanol or *n*-hexane, 50 mL) in a ratio of 1:10 in a flask, and subjected to ultrasound treatment (Bandelin Sonorex Digitec DT 103H, Berlin, Germany) at two temperatures (room temperature of 22 °C and 55 °C, with the elevated temperature selected based on Castejón et al. [7]) for 15 min. Next, the extract was filtered (Frisenette ApS grade 202, Frisenette, Knebel, Denmark) and evaporated in a rotary evaporator (R-200, B-490, V-710 Büchi, Flawil, Switzerland) under reduced pressure at 50 °C and dried under an argon stream until constant weight. The oil content was determined gravimetrically and expressed as a percent (*w*/*w*) of dry weight.

#### 3.3.2. Cold Pressing

Cold pressing was performed in a laboratory prototype apparatus (HP 5M, H. Fischer & Co Norf) at Koželj, a company from Dob, Slovenia (pumpkin seeds), while oil extraction was performed at Pečarič, a company from Metlika, Slovenia (linden, marigold, linseed, poppy, and apricot seeds).

#### 3.3.3. Supercritical CO_2_ Extraction

The extraction was performed at Škrlj, a company from Črniče, Slovenia, using the Waters BBES 2.0 supercritical extraction system (Waters, Milford, MA, USA) equipped with a 10 L extraction vessel and three 2 L collection vessels. Samples were extracted using the following extraction parameters: T = 40 °C, *p* = 300 bar, Θ = 120 g CO_2_/min, t = 16 h. The mixture of supercritical CO_2_ and extract was separated into three consecutive collection vessels (C-V), referred to as C-V1, C-V2 and C-V3, with separation temperature conditions of 40, 30, and 30 °C and separation pressure conditions of 150, 70, and 50 bar, respectively. Low temperatures were selected to avoid the thermal degradation of unsaponifiable matter, as suggested by Alverz-Henao et al. [17]. Extractions were completed within four hours, but were carried out over a longer period to maximize yields.

#### 3.3.4. Re-Extraction

Where oilseed residue was available, extraction using hexane was performed with the oilseed cake after cold pressing (pumpkin, linden, and marigold seed cakes) and with meals after extraction with supercritical CO_2_ (linseed, linden, poppy, apricot, and marigold seed meals) to compare oil residuals.

### 3.4. Saponification

Unsaponifiable matter was determined gravimetrically using the modified Ph. Eur. method [54]. A total of 5 g of seed oil was saponified with 50 mL of 2 M KOH in ethanol (96%), while boiling and stirring the sample for 1 h. After cooling, the sample was transferred to a separating funnel with 100 mL of purified water and extracted three times with 100 mL of diethyl ether. The ether extracts were pooled into a separating funnel and washed with purified water (40 mL two times), followed by one wash of 40 mL KOH in water (30 g/L), and then repeatedly washed with purified water (40 mL) until a neutral phenolphthalein reaction occurred. The wash water was then removed and the organic phase was dried in a rotary evaporator (R-200, B-490, V-710 Büchi, Switzerland) under reduced pressure at 40 °C, until a constant mass was achieved. The experiment was performed in triplicate.

### 3.5. Preparation of Silylated Unsaponifiable Matter

For GC–MS analysis, the extracted unsaponifiable matter was silylated to trimethylsilyl (TMS) derivatives to improve the peak shape, resolution, sensitivity, and thermal stability [18,28]. The unsaponifiable fraction (5 mg) was silylated with 50 µL of the silylating reagent I, shaken manually until the sample was dissolved, and centrifuged for 5 min at 13,000 rpm. The reagent was a prepared silylating mixture of hexamethyldisilazane (HMDS), trimethylchlorosilane (TMCS), and pyridine (3:1:9). Afterwards, the mixture was heated in a water bath at 60 °C for 30 min and cooled, and 950 µL of GC–MS-grade *n*-hexane was added and centrifuged again (5 min, 13,000 rpm). The supernatant was dissolved in *n*-hexane in a ratio of 1:100 and transferred to the GC–MS analysis vial.

### 3.6. Preparation of Fatty Acid Methyl Esters (FAMEs)

Fatty acids in oil samples were converted to FAMEs using a methylation method suggested by Salimon et al. [55]. Briefly, 50 mg of an oil sample was mixed with 2 mL of GC–MS-grade *n*-hexane and 1 mL of 2 M methanolic KOH, followed by 30 s of vigorous shaking. Afterwards, samples were boiled for 2 min at 70 °C, 1.2 mL of 1 M HCl was added for neutralization, and the mixture was gently stirred. Immediate phase separation occurred. Another 1 mL of *n*-hexane was added and mixed gently, and the upper phase was transferred to the GC–MS analysis vial.

### 3.7. GC–MS Conditions

GC–MS analyses were performed using a GCMS-QP2010 Ultra system (Shimadzu Corporation, Kyoto, Japan) with an Rxi-5Sil MS column, 30 m × 0.25 mm i.d., 0.25 μm film thickness (Restek Corporation, Bellefonte, PA, US). The temperature program for FAMEs samples was 40 °C → 300 °C (3 °C/min), a hold at 300 °C for 5 min (total analysis time was 91.7 min), and a split ratio of 1:100. A five-minute solvent delay was used. The temperature program for silylated samples was 50 °C → 270 °C (18.3 °C/min), a hold at 270 °C for 55 min (total analysis time was 67 min), and a split ratio of 1:100. A 15 min solvent delay was used. For both procedures, T_inj_ of 250 °C, T_transf.line_ of 300 °C, and T_ion source_ of 200 °C were used, the injection volume was 1 μL, the carrier gas was He (99.99%), and the gas flow was 1 mL/minute. MS conditions included electron impact (EI) mode at an ionization voltage of 70 eV. The full scan was recorded in the mass range of 40–400 *m*/*z* with a scanning frequency of 5 Hz.

### 3.8. GC–MS General Measurement Procedures

Each sample was analyzed in triplicate and the average response was taken for further examination. Compounds were first identified by comparing their mass spectra and retention times to the spectra from NIST11 and FFNSC2 spectral libraries, and then their identity was confirmed by comparing with the mass spectra and retention times of reference compounds. Calibration curves were made for the quantification of campesterol, stigmasterol, β-sitosterol, squalene (two calibration curves due to the wide concentration range), and cholesterol (Table 6).

### 3.9. Antioxidant Activity Assay

The in vitro antioxidant activity of unsaponifiable matter was evaluated using the DPPH (2,2-diphenyl-1-picrylhydrazyl) method adopted from Wagemaker et al. [56] with slight modifications. The unsaponifiable matter of each plant oil was solubilized in DMSO (1 mg/mL), added to 0.04 mg/mL DPPH methanolic solution (2 mL of DPPH solution and 0.5 mL of a sample), mixed to a homogenous solution, and incubated for 30 min in the dark while shaking. The absorbance was measured at 517 nm and was calculated as follows:

AA% = [(A_DPPH_ − A_SAMPLE_)/A_DPPH_] × 100,

where AA represents the antioxidant activity, A_DPPH_ represents the absorption of the DPPH solution, and A_SAMPLE_ represents the absorption of the unsaponifiable matter.

### 3.10. Evaluation of Processes and Statistical Analysis

The evaluation of experimental processes included the evaluation of the silylation procedure and GC–MS method. The repeatability of silylation was carried out by performing three independent silylation reactions, which resulted in RSD values below 10%; repeatability was considered acceptable and the results of silylation were considered reliable. The repeatability and linearity of the GC–MS method were carried out for standards by performing three independent analyses, which resulted in RSD values < 5% and an R^2^ of 0.99, which was considered acceptable. Statistical analyses were performed using the Office Home Excel software. Correlation was presented using the Pearson coefficient, where a significance level of *p* < 0.05 was used.

## 4. Conclusions

Compared to conventional solvent-based and cold-pressed processes, SFE-CO_2_ offers better selectivity, higher yields, and environmental benefits. This technique, which avoids the use of harmful organic solvents such as hexane, offers a safer and more efficient approach to oil extraction while maintaining the integrity of heat-sensitive bioactive compounds. Our study demonstrated that SFE-CO_2_ achieved higher yields of unsaponifiable matter in four out of six plant seeds, particularly in pumpkin and marigold, where high antioxidant activity was also observed. Additionally, SFE-CO_2_ was able to enhance the concentration of key bioactive compounds, such as squalene and γ-tocopherol, compared to cold pressing. However, determining the most suitable extraction method is complicated and requires the careful evaluation of the desired properties of the final product.

Further research is needed to optimize the operating parameters of SFE-CO_2_ extraction to increase efficiency and yield and to achieve the targeted extraction of the desired compounds. Specifically, future studies should focus on optimizing the extraction of unsaponifiable matter and fatty acids, as well as investigating extraction kinetics, to better understand the process dynamics. The wider application of SFE in industry could lead to a move away from traditional hexane-based methods, thereby meeting environmental and health-oriented goals. With continued optimization, SFE has the potential to revolutionize the production of vegetable oils and provide a sustainable and efficient alternative for various industries such as food, cosmetics, and pharmaceuticals.

## Figures and Tables

**Table 1:** Oil yields (%) obtained using different extraction methods. Values represent the percentage of extracted oil relative to the total seed weight (g of extracted oil/g of seeds × 100), where “/” means no extract was obtained and “-” means no data were provided by the manufacturer. Numbers in bold represent the highest yields. Methods include solvent extraction with hexane and ethanol (at 22 °C and 55 °C), cold pressing, and supercritical CO_2_ extraction (C-V1, C-V2, C-V3).

Plant	Solvent Extraction Yield				Cold Pressing Yield (%)	CO_2_ Extraction Yield			
	Hexane (%)		Ethanol (%)						
	22 °C	55 °C	22 °C	55 °C		C-V1 (%)	C-V2 (%)	C-V3 (%)	Total
Pumpkin	**24.6**	22.3	22.3	23.6	10.7	12.7	5.1	1.0	18.8
Flaxseed	10.2	10.7	10.2	11.0	-	26.8	/	/	**26.8**
Linden	10.8	11.2	4.0	9.0	9.7	16.3	/	/	**16.3**
Poppy	29.0	28.7	28.3	28.2	20.0	19.3	15.4	1.9	**36.6**
Apricot	28.1	**31.1**	23.4	24.8	25.0	13.0	8.8	/	21.8
Marigold	8.6	7.8	6.8	7.0	8.3	7.1	6.7	/	**13.7**

**Table 2:** Composition of fatty acids in extracted oils as determined by gas chromatography–mass spectrometry (GC–MS). Values are expressed as relative area percentages (%) of total chromatographic peaks, with an area greater than 0.1%. Fatty acids are listed by common and chemical names. Data are shown for oils extracted using cold pressing (CP), hexane solvent extraction (H), and supercritical CO_2_ extraction (C-V1, C-V2, C-V3). The compounds were identified on the basis of their mass spectra and retention times.

	Oil extraction Method	Total Saturated Fatty Acids	Total Unsaturated Fatty Acids	C14:0 Myristic Acid	C16:0 Palmitic Acid	C17:1 (Z)−10-Heptadecenoic Acid	C18:0 Stearic Acid	C18:1 Malvalic Acid	C18:2 Linoleic Acid	C18:3 A-Linolenic Acid	C18:1 Oleic Acid	C18:1 (Z)-Octadecenoic Acid	C19:1 Sterculic Acid	C19:1 (E)−10-Nonadecenoic	C18:3 8Z,10E,12Z-Linolenic Acid	C18:3 9Z,11E,13E Oktadecatrienic Acid	C20:1 Gordoic Acid	C20:0 Arachidic Acid	C22:0 Behenic Acid	C24:0 Lignoceric Acid
RT				45.11	52.09	54.43	58.44	56.57	57.44	57.57	57.62	57.74	59.55	60.85	62.19	62.97	63.47	64.25	69.63	74.62
Pumpkin	CP	19.2	80.2	0.1	9.8	-	8.8	-	35.4	-	43.8	1.0	-	-	-	-	-	0.4	0.1	0.1
	C-V1	18.4	81.2	0.1	10.4	-	7.6	-	34.6	-	45.5	1.1	-	-	-	-	-	0.3	-	-
	C-V2	18.3	81.0	0.1	11.1	-	6.9	-	35.2	-	44.8	1.0	-	-	-	-	-	0.2	-	-
	C-V3	24.9	71.9	0.3	15.3	-	9.1	-	32.6	-	37.9	1.4	-	0.1	-	-	-	0.2	-	-
	H	19.6	79.8	0.1	9.9	-	9.1	-	35.2	-	43.6	1.0	-	-	-	-	-	0.5	0.1	0.1
Linseed	CP	10.1	89.8	-	5.4	-	4.5	-	14.3	73.9	-	1.4	-	-	-	-	0.1	0.1	-	-
	C-V	9.2	90.6	-	5.5	-	3.7	-	14.0	75.1	-	1.5	-	-	-	-	-	-	-	-
	H	10.5	89.3	-	5.7	-	4.6	-	14.8	73.0	-	1.4	-	-	-	-	0.1	0.1	0.1	-
Linden	CP	9.7	89.8	0.1	8.0	0.7	1.6	5.6	55.0	-	21.4	2.9	4.0	-	-	-	-	0.1	-	-
	C-V	9.4	90.0	-	7.7	0.6	1.6	5.3	54.2	-	23.1	2.8	3.8	-	-	-	-	0.1	-	-
	H	8.8	91.1	0.1	7.3	0.6	1.3	6.0	54.9	-	21.6	2.5	4.9	0.5	-	-	0.1	0.1	-	-
Poppy	CP	11.4	88.6	-	8.6	-	2.7	-	68.4	-	18.6	1.5	-	-	-	-	-	0.1	-	-
	C-V1	10.3	89.1	-	7.5	-	2.7	-	68.5	-	19.1	1.5	-	-	-	-	-	0.1	-	-
	C-V2	10.8	88.7	-	8.4	-	2.4	-	68.9	-	18.3	1.5	-	-	-	-	-	0.1	-	-
	C-V3	17.0	78.6	1.1	12.4	-	3.5	-	54.1	-	22.1	2.3	-	0.1	-	-	-	-	-	-
	H	11.6	88.3	-	8.8	-	2.8	-	68.1	-	18.6	1.5	-	-	-	-	-	0.1	-	-
Apricot	CP	5.7	94.3	-	4.2	0.1	1.4	-	22.2	-	69.5	2.1	-	-	-	-	-	0.1	-	-
	C-V1	5.4	94.1	-	4.1	0.1	1.2	-	22.5	-	69.1	2.0	-	-	-	-	0.1	0.1	-	-
	C-V2	5.6	93.7	-	4.5	0.1	1.1	-	22.8	-	68.3	2.0	-	-	-	-	-	0.1	-	-
	H	5.3	94.6	-	3.9	0.1	1.4	-	20.0	-	72.3	2.0	-	-	-	-	-	-	-	-
Marigold	CP	4.6	95.0	-	2.8	-	1.5	-	28.7	-	6.1	0.6	-	-	58.1	0.8	0.4	0.4	-	-
	C-V1	4.3	94.7	-	2.3	-	1.6	-	25.8	-	4.3	0.6	-	-	62.2	1.2	0.3	0.4	-	-
	C-V2	4.8	93.9	-	2.8	-	1.7	-	27.6	-	5.2	0.6	-	-	58.9	0.8	0.3	0.4	0.1	-
	H	5.9	90.7	-	3.3	-	2.0	-	26.9	-	4.9	0.6	-	-	56.3	1.4	0.4	0.4	0.1	0.1

**Table 3:** Yield and composition of the unsaponifiable matter and antioxidant activity of extracted oils. Results are shown for oils extracted via cold pressing (CP) and supercritical CO_2_ extraction (C-V1, C-V2, C-V3). Unsaponifiable matter is expressed as a percentage (%) of the total oil weight. The composition of unsaponifiable matter was analyzed by GC–MS and is presented as the relative area percentage (%) of chromatographic peaks, with an area greater than 0.1%. Antioxidant activity was measured using the DPPH assay and expressed as inhibition percentage. RSD represents the repeatability of the extraction method for isolating the unsaponifiable matter. The compounds were identified on the basis of their mass spectra and retention times.

		Pumpkin				Linseed		Linden		Poppy				Apricot			Marigold		
Oil extraction method		CP	C-V1	C-V2	C-V3	CP	C-V	CP	C-V	CP	C-V1	C-V2	C-V3	CP	C-V1	C-V2	CP	C-V1	C-V2
% total unsaponified matter		1.1	1.2	1.5	4.0	1.4	1.7	1.4	2.0	0.7	0.7	0.9	1.3	0.7	0.8	1.5	1.7	2.2	2.2
RSD (unsaponified matter extraction)		13.4	0.1	27.9	0.9	0.6	25.9	19.5	0.1	19.9	20.5	14.5	9.5	16.3	0.3	10.7	8.5	2.4	1.2
compound	RT	**relative percentage of area** (**%**)																	
squalene	19.40	50.6	29.5	65.1	76.9	-	0.2	2.2	2.2	0.2	-	0.2	0.9	2.3	0.5	4.9	0.6	-	0.6
γ-tocopherol	23.37	2.8	2.7	1.5	1.9	1.4	0.3	1.9	2.4	-	-	-	-	1.0	0.2	0.4	7.5	4.1	18.8
cholesterol	28.23	-	4.6	-	-	-	-	-	-	-	-	-	-	-	-	-	1.2	-	1.3
desmosterol	32.12	-	-	-	-	-	-	-	-	0.9	-	0.9	0.6	-	-	-	-	-	-
campesterol	32.48	-	-	-	-	11.9	13.9	4.8	4.4	12.7	15.3	10.8	17.0	2.2	0.9	1.7	5.7	5.1	6.1
stigmasterol	33.64	-	-	-	-	3.7	2.7	2.8	2.6	1.6	1.8	1.7	1.6	-	-	-	11.8	11.7	14.0
β-sitosterol	36.76	23.7	19.6	19.6	12.3	32.9	20.1	67.3	81.4	54.3	41.7	40.9	54.1	83.7	90.1	62.1	34.7	50.9	33.4
lanosterol	38.22	-		-	-	-	-	-	-	-	-	-	-	-	-	-	2.8	-	-
cycoartenol	39.33	12.3	4.2	8.3	5.3	-	-	1.4	-	-	-	-	-	-	-	-	-	-	-
cholest-7-en-3-ol	40.44	4.0	4.6	-	-	-	-	1.1	-	-	1.6	1.7	1.9	-	-	-	4.7	11.4	4.8
9,19-cyclolanost-24-en-3-ol	40.69	-	-	-	-	26.1	22.1	-	-	-	-	-	-	-	-	-	-	-	-
unknown component (*m*/*z*: 343 (100) 75 (57) 55 (52) 73 (46))	41.40	6.6	11.3	5.5	3.6	-	-	-	-	7.9	-	5.7	-	-	-	-	-	-	-
24-methylene-9,19-cyclolanostan-3-ol	44.45	-	-	-	-	6.3	1.7	-	-	-	-	0.5	-	-	-	-	-	-	-

**Table 4:** Quantification of selected unsaponifiable matter compounds in extracted oils. Results are provided for oils obtained via cold pressing (CP) and supercritical CO_2_ extraction (C-V1, C-V2, C-V3). Data are presented as mg of compound per g of oil (mg/g), determined by GC–MS analysis. Quantified compounds include squalene, β-sitosterol, campesterol, stigmasterol, and cholesterol, using calibration curves with reference standards.

		Pumpkin				Linseed		Linden		Poppy				Apricot			Marigold		
	Oil Extraction Method	CP	C-V1	C-V2	C-V3	CP	C-V	CP	C-V	CP	C-V1	C-V2	C-V3	CP	C-V1	C-V2	CP	C-V1	C-V2
Compound	RT	mg/g																	
Squalene	19.40	0.07	0.05	0.07	0.13	-	0.50	0.04	0.04	0.51	-	0.38	0.44	0.04	0.03	0.04	0.39	-	0.22
Cholesterol	28.23	-	-	-	-	-	-	-	-	-	-	-	-	-	-	-	0.13	-	0.17
Campesterol	32.48	-	-	-	-	2.44	2.18	2.05	1.48	1.93	1.34	3.35	2.87	0.71	0.26	0.84	0.85	0.38	1.21
Stigmasterol	33.64	-	-	-	-	1.39	0.78	2.31	1.65	0.42	0.27	1.10	0.59	-	-	-	3.42	1.33	1.71
β-sitosterol	36.76	2.77	2.83	2.58	2.65	2.90	2.68	4.20	4.08	2.98	2.71	3.73	3.12	4.02	3.67	4.28	2.81	2.69	3.07

**Table 5:** List of plants with the origins and suppliers of the seeds used for oil extraction.

Common Plant Name	Latin Plant Name	Source	Seed Origin
Pumpkin	*Cucurbita pepo* L.	Medilip d. o. o.	Slovenia
Linseed	*Linum usitatissimum* L.	Oil-extraction Pečarič	Hungary
Linden	*Tilia* sp.	Vilmorin	France
Poppy	*Papaver somniferum* L.	Oil-extraction Pečarič	Turkey
Apricot	*Prunus armeniaca* L.	Oil-extraction Pečarič	Turkey
Marigold	*Calendula officinalis* L	Fosters Seeds	USA

**Table 6:** Reference compounds and calibration curves for GC–MS quantification. Values represent the linear equations of standard curves (y = peak area, x = concentration in μg/mL), along with their concentration ranges and coefficients of determination (R^2^).

Standard	Curves	Range (μg/mL)	R^2^
Cholesterol	y = 625,175x − 626,279	0.58–5.83	0.996
Campesterol	y = 122,204x − 80,396	0.50–4.99	0.993
Stigmasterol	y = 60,509x + 20,006	0.50–5.04	0.989
β-sitosterol	y = 2 × 10^6^x − 5 × 10^7^	22.68–170.10	0.990
Squalene	y = 6 × 10^7^x − 2 × 10^7^	34.6–173.0	0.990
Squalene	y = −54,342x + 316,939	0.35–3.01	0.991

## Data Availability

Data are contained within the article.
